# Flexible Pressure Sensor with Tunable Sensitivity and a Wide Sensing Range, Featuring a Bilayer Porous Structure

**DOI:** 10.3390/mi16040461

**Published:** 2025-04-13

**Authors:** Yunjiang Yin, Yingying Zhao, Tao Xue, Xinyi Wang, Qiang Zou

**Affiliations:** 1 School of Microelectronics, Tianjin University, Tianjin 300072, China; 2022232158@tju.edu.cn; 2 Tianjin Flying Pigeon Group Co., Ltd., Tianjin 301600, China; 18622912991@163.com (Y.Z.); 13116091576@163.com (X.W.); 3 Center of Analysis and Testing Facilities, Tianjin University, Tianjin 300072, China; xuetao@tju.edu.cn; 4 Tianjin International Joint Research Center for Internet of Things, Tianjin 300072, China; 5 Tianjin Key Laboratory of Imaging and Sensing Microelectronic Technology, Tianjin University, Tianjin 300072, China; 6 State Key Laboratory of Advanced Materials for Intelligent Sensing, Tianjin 300072, China

**Keywords:** flexible piezoresistive pressure sensors, wearable electronics, bilayer porous structure, tunable

## Abstract

Flexible piezoresistive pressure sensors have great potential in wearable electronics due to their simple structure, low cost, and ease of fabrication. Porous polymer materials, with their highly deformable internal pores, effectively expand the sensing range. However, a single-sized pore structure struggles to achieve both high sensitivity and a broad sensing range simultaneously. In this study, a PDMS-based flexible pressure sensor with a bilayer porous structure (BLPS) was successfully fabricated using clamping compression and a sacrificial template method with spherical sucrose cores. The resulting sensor exhibits highly uniform pore sizes, thereby improving performance consistency. Furthermore, since different pore sizes and thicknesses correspond to varying Young’s moduli, this study achieves tunable sensitivity across a wide pressure range by adjusting the bilayer thickness ratio (maximum sensitivity of $0.063\;{\mathrm{kPa}}^{-1}$ in the 0–23.6 kPa range, with a pressure response range of 0–654 kPa). The sensor also demonstrates a fast response time (128 ms) and excellent fatigue stability (>10,000 cycles). Additionally, this sensor holds great application potential for facial expression monitoring, joint motion detection, pressure distribution matrices, and Morse code communication.

## 1. Introduction

Flexible pressure sensors have attracted considerable attention in fields such as wearable devices [1,2,3,4,5,6,7], electronic skin [3,8,9,10,11,12,13], body health monitoring [14,15,16,17], and artificial intelligence [18,19,20] due to their high sensitivity [21,22,23], excellent flexibility [22,24], and lightweight nature [24]. These sensors are frequently employed to monitor various human body movements, such as swallowing [25], joint bending (e.g., fingers and knees) [2,26], and footpad compression [22,27,28]. In diverse applications, a major challenge lies in maintaining high sensitivity [29] over a wide pressure range [30], which is crucial for the practical application of flexible pressure sensors.

The sensing mechanisms of commonly used flexible pressure sensors can be categorized into three types: piezoresistive [1], piezoelectric [31], and capacitive [32]. Among these, piezoresistive sensors are particularly popular due to their simple structure, ease of measurement, low cost [33], and straightforward fabrication methods [34]. Polymer porous materials [2], such as thermoplastic polyurethane (TPU) [17,35] and polydimethylsiloxane (PDMS) [35,36], are often employed as sensing substrates due to their wide compression range. Moreover, these materials can adsorb conductive substances, such as conductive fillers (e.g., carbon black (CB) [36,37], reduced graphene oxide (rGO) [38,39], or carbon nanotubes (CNTs) [32]) on their surfaces, potentially leading to enhanced sensitivity and broader sensing ranges, making them widely used in piezoresistive sensors.

Currently, flexible porous piezoresistive sensors face two significant issues. One issue is that the pore shape and size of the sensing substrate are not uniform. Recently, Ye Tian et al. [40] used a template-removal method based on sugar cubes, and the resulting sensor achieved a sensitivity of $3.54\;{\mathrm{kPa}}^{-1}$ within a sensing range of less than 8 kPa, with a broader sensing range of 0–150 kPa. However, the pores of the sensor’s sensing layer were not uniform, leading to performance variations among the fabricated sensors. Wei Li et al. [41] improved the uniformity of the sensor’s pores by grinding, sieving, and dissolving NaCl particles. The sensors made using this method showed improved pore uniformity and a sensitivity of $10.805\;{\mathrm{kPa}}^{-1}$ in the 0.001–1 kPa pressure range. However, due to the non-uniform shape of the crystals, performance variations still exist between individual sensors. Although these sensors can be used in sensor matrices [9,42], they undoubtedly increase the difficulty of subsequent calibration and debugging. Another issue is that porous flexible pressure sensors with a single pore size struggle to achieve both a wide sensing range and high sensitivity simultaneously. Yi-Fan Yang et al. [38] formed a pore size gradient by incorporating multi-layer composites with different pore sizes and integrated them into a single sensor. This design demonstrated high sensitivity ($1.412\;{\mathrm{kPa}}^{-1}$) within a pressure range of 1275 kPa. The study indicated that the multi-layer structure based on pore size plays a significant role in modulating the performance of the sensor.

In this study, we successfully developed a flexible, dual-layer porous pressure sensor based on PDMS using a simple clamp-pressing method and a sacrificial template method with sucrose sugar balls as the sacrificial material. Two layers of CNTs/PDMS sponges with different thicknesses and pore sizes are integrated to form a flexible pressure sensor with a BLPS. The spherical sucrose sugar balls used as the sacrificial layer ensure the uniformity of the pore sizes, while the clamp-pressing method ensures the thorough dissolution of the sacrificial layer, further enhancing the performance consistency of sensors with the same specifications across different individuals. In the dual-layer porous structure, with the sensor thickness fixed, the dual-layer porous PDMS pressure sensor achieves a wide detection range and adjustable high sensitivity by varying the thickness ratio between the large-pore and small-pore layers. The sensor demonstrates a maximum sensitivity of $0.063\;{\mathrm{kPa}}^{-1}$ within the 0–23.6 kPa range, and a pressure response range of up to 0–654 kPa. The sensor also demonstrates a remarkably fast response time (128 ms), recovery time (158 ms), and excellent stability (>10,000 stable cycles). Moreover, the PDMS material itself has excellent flexibility and compressibility, allowing it to adapt to pressure variations on different shapes and surfaces and achieve a perfect fit with the human body. With these outstanding properties, this pressure sensor can be widely applied in various scenarios, including pressure/weight monitoring, facial micro-expression detection, joint motion detection, human–machine matrix interaction, and Morse code communication.

## 2. Materials and Methods

### 2.1. Materials and Chemicals

Multi-walled carbon nanotubes (CNTs), with a length of 5–15 µm, a diameter of 10–30 nm, and purity of ≥96%, and carbon nanotube dispersant were purchased from Jiangsu Xianfeng Nano Materials Technology Co., Ltd. (Nanjing, China). The sucrose core particles were purchased from Haining Weijing Pharmaceutical Excipients Technology Development Co., Ltd. (Jiaxing, China). PDMS Sylgard 184 was purchased from Dow Corning Corporation (Midland, Michigan). Sodium dodecyl sulfate ($C_{12}H_{25}O_{4}NaS$) was purchased from Shanghai Macklin Biochemical Technology Co., Ltd. (Shanghai, China). The stretchable silver paste was purchased from Shenzhen Yilai Technology Co., Ltd. (Shenzhen, China). All chemicals were used as received without further purification.

### 2.2. Preparation of BLPS Sponge

**Figure 1:**
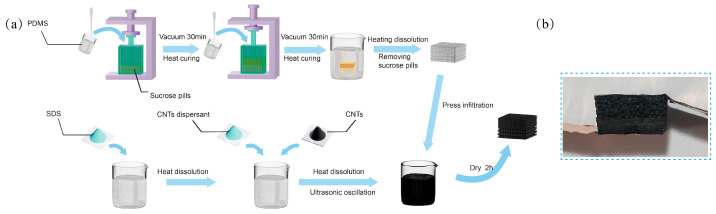
Preparation process and actual sample of BLPS sensors: (**a**) Preparation process of BLPS sensors. (**b**) Actual sample of BLPS sensor.

First, the small (0.4 mm) sucrose sugar spheres were placed into a prepared square mold, and pressure was applied to both ends using a clamp to ensure sufficient contact between the sugar spheres, as shown in Figure 1a. Next, the prepared PDMS prepolymer and curing agent were thoroughly mixed in a 10:1 mass ratio and poured into the mold. The mold and sample were then placed in a vacuum chamber, and a vacuum was applied for 2 h to ensure that the gaps between the sucrose spheres were filled with PDMS. Afterward, the mold was placed in a 60 °C oven for 6 h to fully cure, thereby completing the first layer. Then, the larger sucrose sugar spheres (1 mm) were placed on top of the first layer in the mold, and the same procedure used for the first layer was followed. Once the PDMS was cured, the double-layer composite material was removed from the mold. Finally, the resulting composite material was subjected to a water bath at 100 °C to remove the sacrificial template (sucrose spheres), thereby obtaining the PDMS sponge with a BLPS structure.

### 2.3. Preparation of CNT Solution

A total of 1 g of sodium dodecyl sulfate (SDS) was dissolved in 98.8 g of deionized water. The solution was maintained at a constant temperature of 60 °C on a heating platform and stirred uniformly using a glass rod to ensure homogeneity. Subsequently, 0.1 g of a CNT dispersant was added and stirred until completely dissolved. Following this, 0.1 g of carbon nanotube (CNT) powder was introduced into the prepared solution. After allowing the CNT powder to settle completely, the mixture was subjected to ultrasonication for 20 min using an ultrasonic oscillator. This process was repeated 4 to 6 times to achieve thorough dispersion of the CNTs within the solution. The resulting dispersion was then cooled to room temperature for 2 h and subsequently filtered through a 300-mesh filter to obtain a CNT solution with a concentration of 0.1%. The final solution was stored in a sealed glass container for subsequent use.

### 2.4. Fabrication of the BLPS Sensor

The BLPS sponge was immersed in a prepared 0.1 Wt% CNTs solution and pressed 15–20 times using a mold, with each compression causing more than 80% deformation of the sponge to ensure full infiltration of the CNT solution into the sponge. After standing at room temperature in the solution for 3 h, the sponge was removed and placed in a 60 °C vacuum oven to dry for 2 h. Then, a stretchable silver paste was evenly applied to both sides of the porous PDMS composite material to adhere copper sheet electrodes, which were dried in a 60 °C vacuum oven for 2 h. Finally, the fabrication of the double-layer porous composite flexible pressure sensor was completed. A physical diagram of the BLPS sensor after copper electrode encapsulation is shown in Figure 1b.

### 2.5. Characterization and Measurements

Scanning electron microscopy (SEM, Regulus 8100, Hitachi, Beijing, China)and optical microscopy (Oplenic Digital Camera Oplenic, Hangzhou, China) were used to capture the surface morphology of the dual-layer porous PDMS composite material. Pressure was applied using a force gauge (AIGU-ZP-1000, AIGU, Shenzhen, China) in combination with a self-assembled, high-precision displacement meter setup, with testing conducted at room temperature. The resistance changes in the sensor were measured using an LCR meter (Keithley 2110, Tektronix, Shanghai, China). A 9V voltage for the LED light bulb was supplied by a DC power supply (KXN-305D, ZHAOXIN, Shenzhen, China).

## 3. Results

### 3.1. Physical Structure Characterization

**Figure 2:**
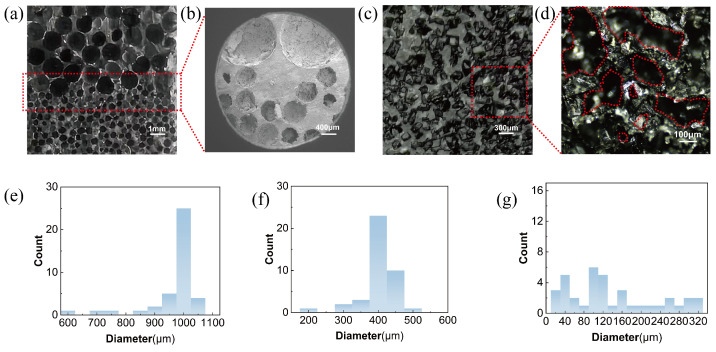
Microstructure images of the BLPS sensor with a bilayer structure and the sensor fabricated using the sucrose method, along with the pore size histograms: (**a**) Bilayer structure under an optical camera. (**b**) SEM image of the bilayer structure. (**c**) Sensor fabricated using the sucrose method under an optical camera. (**d**) Magnified structure of the sensor fabricated using the sucrose method under an optical microscope. (**e**) Histogram of pore sizes in the large-pore region of this study. (**f**) Histogram of pore sizes in the small-pore region of this study. (**g**) Histogram of pore sizes in the sugar cube method.

Figure 2a presents the microscopic morphology of the BLPS sensor captured using an optical camera. As observed in the image, the pore sizes within each layer of the dual-layer structure are uniformly distributed and densely packed. The region highlighted by the red box marks the interface between the two layers, where the tight connection and the absence of visible gaps further confirm the excellent adhesion between the layers, indicating the outstanding mechanical stability of the sensor.

Figure 2b shows an enlarged view of the BLPS sensor under SEM at the demarcation line, taken from a different side of the same sensor as in Figure 2a, further showing the uniformity of the sample’s pore size. Under the same experimental conditions, sensors fabricated using the sugar cube template method (Figure 2c,d) exhibit a highly irregular pore size distribution, ranging from 0.02 mm to 0.34 mm, as indicated by the red dashed lines. Figure 2e–g present histograms of the pore size distribution for the large-pore and small-pore layers of the sensor in this study, as well as the sugar cube template-based sensor. It is evident that in the proposed sensor, more than 85% of the pores in the large-pore layer (0.95–1.05 mm) and small-pore layer (0.4–0.45 mm) fall within a narrow size range, whereas the sugar cube template-based sensor exhibits a highly inconsistent pore distribution. These findings demonstrate that the clamp compression and sacrificial template methods significantly enhance pore uniformity, thereby ensuring the stability and reliability of the sensor’s performance.

**Figure 3:**
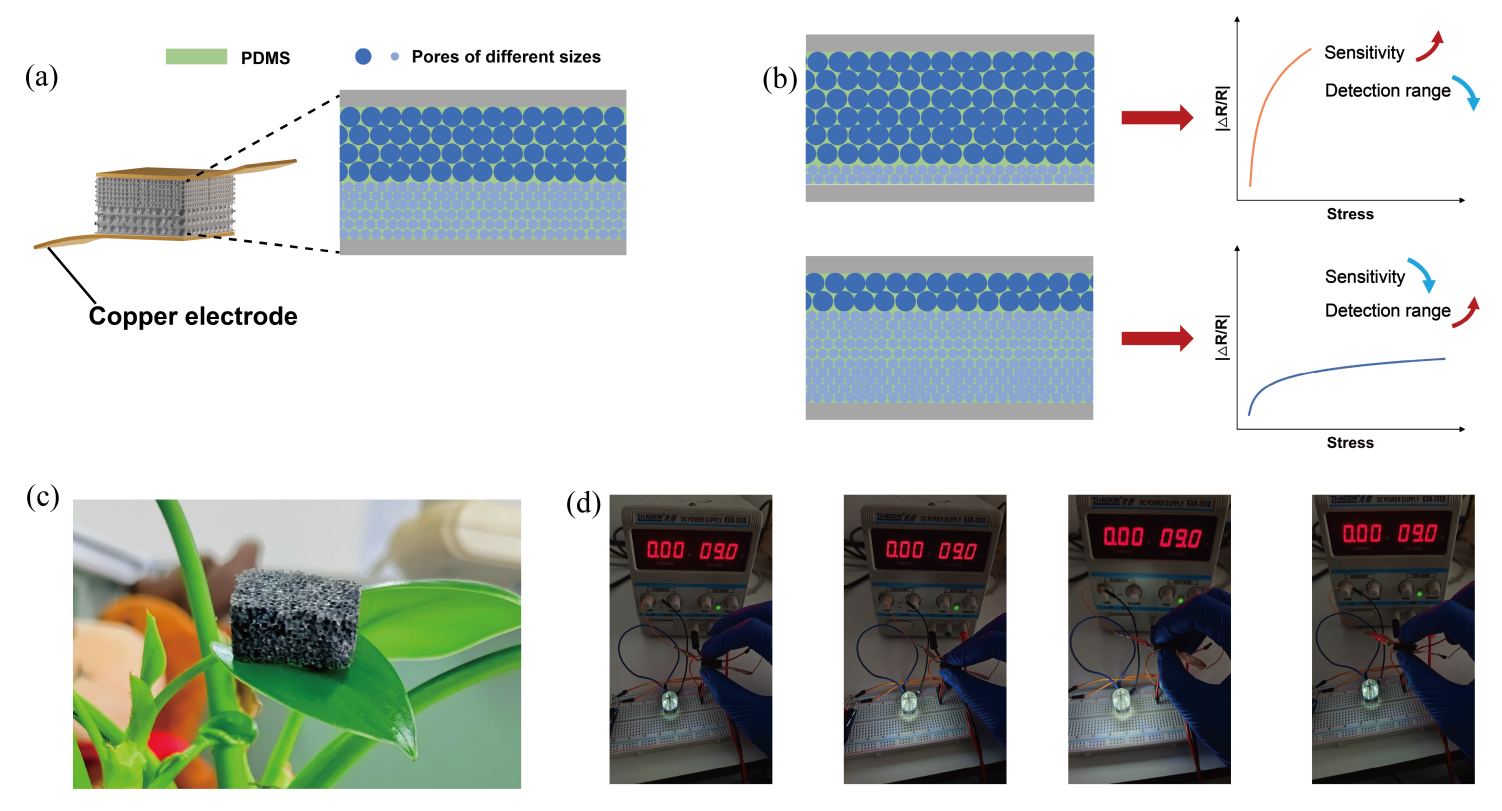
Schematic diagram of the BLPS sensor model and its basic macroscopic physical properties. (**a**) Model diagram of the BLPS sensor. (**b**) Performance design concept of the BLPS sensor. (**c**) The BLPS sensor supported on the green leaves of a pothos without bending. (**d**) Sensor brightness control of LED lights via compression and twisting.

In addition to its excellent characterization at the microscopic level, the BLPS sensor also exhibits good physical properties at the macroscopic level. Figure 3a illustrates the schematic of the sensor, where the top and bottom ends of the BLPS sensor are copper electrodes connected to the middle layer through the stretchable silver paste. The middle layer is composed of a double-layer porous PDMS sponge that is impregnated with a CNT solution and dried. These three layers together form the BLPS sensor with adjustable sensitivity and pressure-sensing range. While maintaining a constant sensor thickness, increasing the thickness ratio of the large-pore layer enhances the sensitivity of the sensor under low stress. Conversely, increasing the thickness ratio of the small-pore layer expands the sensor’s pressure-sensing range, as shown in the performance schematic in Figure 3b. Due to the porous characteristics of BLPS and the excellent elasticity of the PDMS substrate material, the BLPS sensor inherits the superior properties of both materials.

Figure 3c shows that the sensor can be placed on the surface of the green leaves of a pothos plant without bending or deforming the leaves, demonstrating its lightweight nature. This feature provides a solid foundation for its application in wearable devices. Figure 3d demonstrates that the sensor can quickly return to its original state after being compressed and bent, exhibiting excellent compressibility and flexibility. These outstanding properties provide a solid foundation for its application in wearable pressure sensors. Furthermore, by periodically compressing and releasing the sensor, its resistance can be altered, enabling precise control over the brightness of the LED light bulb, further validating its practical application value.

### 3.2. Sensing Mechanism

In general, the wide pressure-sensing range of flexible porous pressure sensors primarily arises from their internally compressible pore structure. Small and dense pores provide greater internal support, which is key to the wide pressure-sensing range of flexible porous-structure pressure sensors. However, small pores struggle to produce sufficient pore deformation under low stress, resulting in lower sensitivity. In contrast, the large-pore structure effectively addresses this issue by generating sufficient deformation under low stress, thereby improving sensitivity. In this study, both pore structures were integrated into the same sensor. By adjusting the thickness ratio between the two pore structures while maintaining the overall sensor thickness, the balance between high sensitivity and a wide pressure-sensing range was successfully achieved.

**Figure 4:**
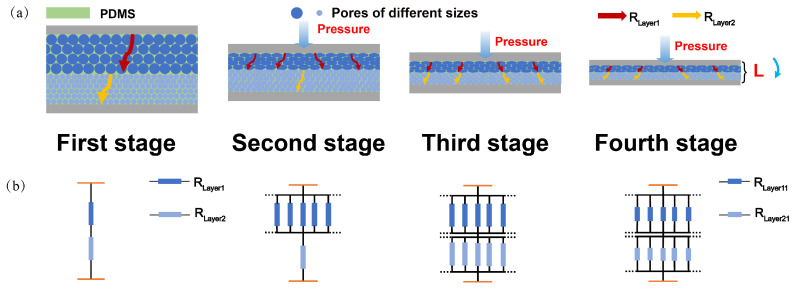
Conductive pathways and resistance equivalent model of the BLPS sensor at different pressure stages: (**a**) Changes in pore size and conductive pathways of the BLPS sensor at different pressure stages. (**b**) Resistance equivalent model of the BLPS sensor at different pressure stages.

The process of pore variation in the sensor can be divided into four main stages. As shown in Figure 4a, in the first stage, when the sensor is not subjected to external pressure, the overall resistance $R_{All}$ is the sum of the inherent resistances of the two porous layers, $R_{layer1}$ and $R_{layer2}$, in series, as expressed by

(1) $$R_{All}=R_{Layer1}+R_{Layer2}.$$

In the second stage, as the vertically applied pressure gradually increases, the deformation is primarily contributed by the large-pore sensing layer. Due to the relatively low Young’s modulus of the large-pore structure, the pore walls gradually close under low stress. Conversely, the small pores, which have a higher Young’s modulus, undergo minimal deformation. The closure of the large pores leads to the formation of a new conductive path. It is assumed that the newly formed Layer1 parallel resistors all have a resistance of $R_{Layer1}$. As a result, the resistance of the large-pore layer decreases, and it transforms into n1 parallel resistors of $R_{Layer1}$, with the overall resistance changing to

(2) $$R_{All}=\frac{R_{Layer1}}{n1}+R_{Layer2}.$$

As the pressure continues to increase, the system enters the third stage. The vertical pressure continues to increase, causing the small-pore layer to gradually close, thereby forming a second conductive path. Similarly, the resistance of the small-pore layer becomes $\frac{R_{Layer2}}{n2}$, and the overall resistance changes to

(3) $$R_{All}=\frac{R_{Layer1}}{n1}+\frac{R_{Layer2}}{n2}.$$

As the external force continues to increase, reaching the fourth stage, the pores of the sensor are completely closed. According to the resistance calculation formula $R=\rho \frac{L}{S}$, as the applied force increases, the sensor’s thickness L continues to decrease. While no new conductive pathways are formed, the resistance of the large-pore layer and the small-pore layer decreases with increasing force, becoming $R_{Layer11}$ and $R_{Layer21}$. As a result, the overall resistance $R_{All}$ becomes

(4) $$R_{All}=\frac{R_{Layer11}}{n1}+\frac{R_{Layer21}}{n2}.$$

At this point, the overall resistance continues to decrease slowly. The specific resistance equivalent model is shown in Figure 4b.

### 3.3. Basic Sensing Characteristics

**Figure 5:**
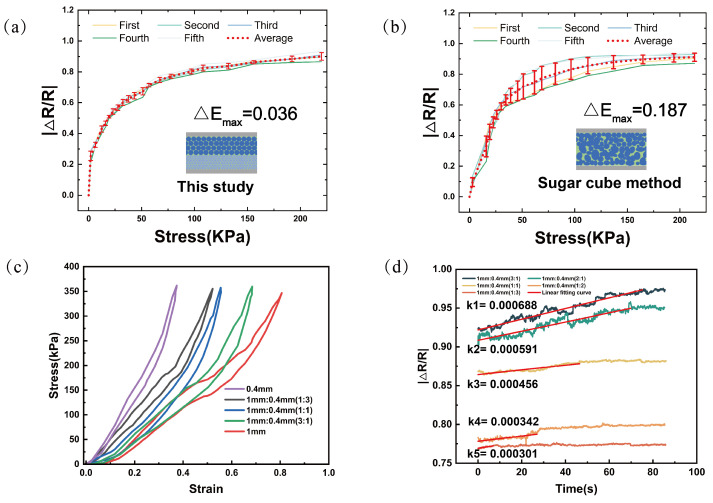
Comparison of the performance errors of five sensors prepared using the method in this study and the sugar cube dissolution method, as well as hysteresis effect curves and creep curves of five sensors with different thickness ratios: (**a**) Performance error of |ΔR/R| for sensors prepared using the method in this study in the 0–223 kPa pressure range. (**b**) Performance error of |ΔR/R| for sensors prepared using the sucrose dissolution method in the 0–220 kPa pressure range. (**c**) Comparison of hysteresis effects. (**d**) Comparison of creep effects.

The BLPS sensors, fabricated using the clamp compression strategy and spherical sucrose cores as sacrificial materials via the sacrificial template method, exhibit excellent performance consistency under identical conditions, as shown in Figure 5a,b.

To compare the performance stability of individual sensors prepared using the sucrose dissolution method, five sensors with the same specifications (10 mm in length and width, 5 mm in height) were fabricated under the same environmental conditions—one using the clamp compression strategy with spherical sucrose cores as sacrificial materials, and the others using the sucrose dissolution method. The results show that the fabrication method employed in this study offers a significant advantage in terms of performance consistency between samples. Under equal pressure, the maximum resistance change rate difference, $\Delta E_{max}$, is only 0.036 for the BLPS sensors, whereas for the sensors made using the sucrose dissolution method, $\Delta E_{max}$ is 0.187, five times greater than that of the BLPS sensors. The superior performance consistency of the BLPS sensors facilitates the easier fabrication of sensor arrays.

Hysteresis and time drift (creep) are important performance metrics for pressure transducers, and the experimental results are shown in Figure 5c,d. Figure 5c demonstrates the stress–strain compression and release tests for sensors with different pore-layer thickness ratios at pressures ranging from 0 to 350 kPa with the motor running at 50 mm/min. It is evident from the graphs that the hysteresis between the pressurization and release curves becomes more pronounced as the large-pore (1 mm) thickness ratio increases. This is specifically reflected in the increasing area enclosed by the hysteresis ring, where the hysteresis rate changes from 12.3% for a thickness ratio of 1 mm:0.4 mm (0:1) to 15.66% for 1 mm:0.4 mm (1:0). Therefore, as the macroporous layer’s thickness ratio increases, Young’s modulus is lower, and the thickness of the sensor varies more at the same pressure. While this improves the sensitivity of the sensor under low-pressure conditions, it also affects the recovery rate during unloading and therefore increases the hysteresis rate.

Figure 5d shows the time drift (creep) of the sensors with different pore-layer thickness ratios, where it can be seen that under a continuous load of 100 kPa, the resistance change rate of the sensors with different thickness ratios increases and eventually stabilizes. This is due to the fact that under a large amount of stress, the internal pore structure of the sensing layer is first compressed instantaneously to a certain deformation and then continues to be compressed slowly. When the support force of the internal pore structure equals the external pressure, the resistance change stabilizes. However, sensors with different thickness ratios have different abilities to resist the subsequent slow compression process due to their different Young’s moduli. As the thickness ratio of the large-pore layer increases, the Young’s modulus of the sensor decreases, and the internal structure of the pore becomes looser and more prone to deformation, so the ability to resist the subsequent slow compression process is weaker, the time required to maintain smoothness is longer, and the slope of the resistance change during the process is larger. In contrast, the internal structure of the small-pore layer is more compact, the ability to resist the subsequent slow compression process is stronger, the time required to maintain smoothness is shorter, and the slope of the resistance change during the process is smaller.

**Figure 6:**
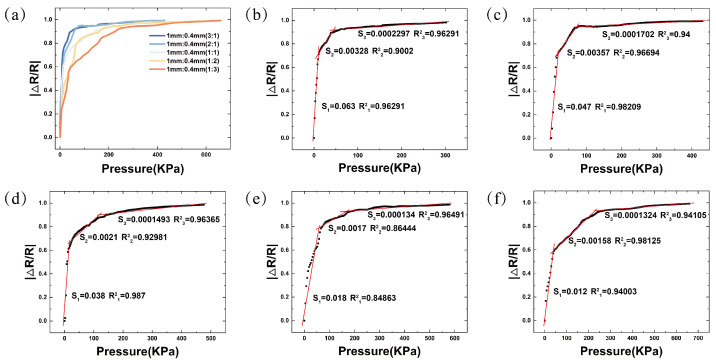
Performance curves of BLPS sensors with the same size (10 mm × 10 mm × 6 mm) at different thickness ratios of 1 mm:0.4 mm pore layers, and the comparison of sensitivity S and variance R^2^ at each stage (The black dashed line in the above figure represents the actual measured data, and the red straight line is the linear fitting result): (**a**) Comparison of the performance curves of |ΔR/R| with pressure for BLPS sensors with five different thickness ratios. (**b**) Thickness ratio of 3:1. (**c**) Thickness ratio of 2:1. (**d**) Thickness ratio of 1:1. (**e**) Thickness ratio of 1:2. (**f**) Thickness ratio of 1:3.

Thanks to the adjustable thickness ratio of the double-layer porous structure, the fabricated sensor maintains high sensitivity while expanding the pressure-sensing range. To further investigate the effect of changing the thickness ratio of different pore layers on the sensor’s performance, under the condition of a constant sensor thickness, five double-layer porous pressure sensors with varying thickness ratios of the pore layers were fabricated in this study. All sensors had dimensions of 10 mm × 10 mm × 6 mm, and the fabrication conditions were consistent with those described in the “Experiment” section. A pressure gauge was used to apply pressure, and an LCR meter was used to measure the resistance change in the sensor. The sensitivity of the flexible porous pressure sensor is defined as $S=\frac{\delta |\Delta R/R|}{\delta P}$, where R is the initial resistance of the sensor in the absence of pressure, ΔR is the change in resistance (i.e., $R_{1}$-R), $R_{1}$ is the resistance of the sensor under applied pressure, and δP is the change in pressure applied to the sensor. Sensitivity can be calculated by determining the slope of the tangent to the resistance change versus the pressure curve. Figure 6a shows that as the thickness ratio of the 0.4 mm pore layer increases, the sensor’s sensitivity decreases, but its pressure-sensing range significantly increases, with the sensor thickness held constant. Specific performance is presented in Figure 6b–f. The data reveal that the BLPS sensor exhibits three linear regions across different pressure ranges. For example, with a thickness ratio of 1:1 for the 1 mm and 0.4 mm layers, the first stage shows a sensitivity of $S_{1}$ = $0.038\;{\mathrm{kPa}}^{-1}$ in the 0–35.4 kPa range, the second stage shows a sensitivity of $S_{2}$ = $0.0021\;{\mathrm{kPa}}^{-1}$ in the 35.4–147.76 kPa range, and the third stage shows a sensitivity of $S_{3}$ = $0.0001493\;{\mathrm{kPa}}^{-1}$ in the 147.76–476.5 kPa range. The corresponding fitting curve variances are $R_{1}^{2}$ = 0.987, $R_{2}^{2}$ = 0.92981, and $R_{3}^{2}$ = 0.96365, exhibiting excellent linearity. Compared to other porous piezoresistive sensors, this sensor maintains high sensitivity while extending the pressure-sensing range. The adjustable thickness ratio characteristic of the double-layer porous structure provides high performance over a wide pressure range, making it highly suitable for various applications, such as human motion monitoring.

**Figure 7:**
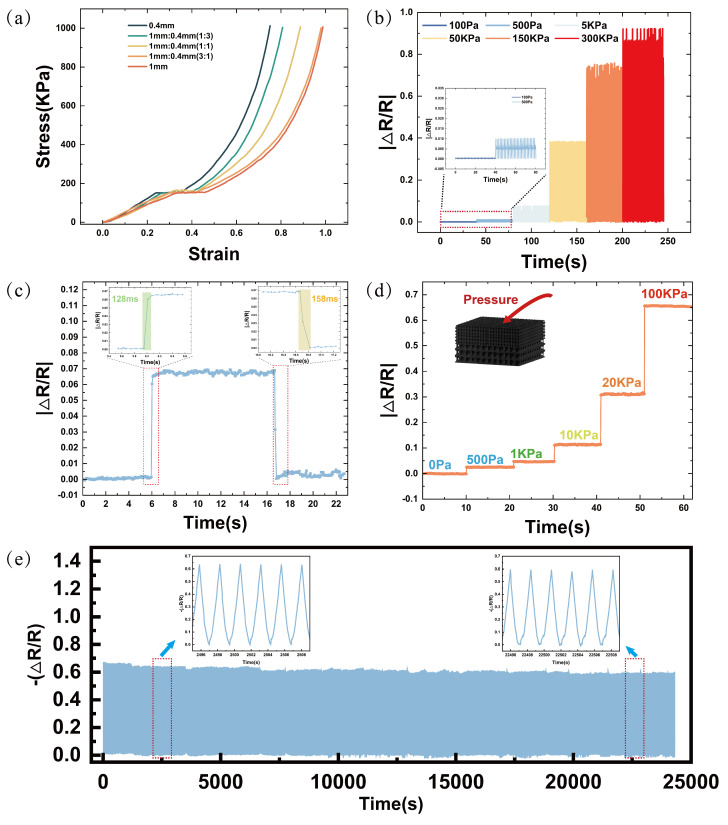
Stress–strain curves of BLPS sensors with different thickness ratios and basic performance at different pressure levels, as well as BLPS sensor fatigue testing: (**a**) Stress–strain curves of BLPS sensors with different thickness ratios of 1 mm and 0.4 mm pore layers. (**b**) Resistance response of BLPS sensors under repeated pressing at different pressure levels. (**c**) Response time and recovery time of the BLPS sensor at 1 kPa pressure. (**d**) Resistance response of the BLPS sensor maintained for 40 s at different pressure levels. (**e**) Fatigue stability test with >10,000 cycles.

The adjustable performance characteristics of BLPS sensors are closely related to the pore size and the thickness ratio of the pore layers. To investigate the effect of the dual-layer porous structure on the sensor’s stress–strain properties, control samples with single-layer and dual-layer porous structures at different thickness ratios were prepared under identical experimental conditions. All samples had the same dimensions (10 mm × 10 mm × 6 mm). Figure 7a illustrates the stress–strain curves of the sensors tested within the 0–1000 kPa range using a compression testing machine. It is evident that as the thickness ratio of the 0.4 mm pore layer increases, the deformation of the sensor decreases under the same pressure. For sensors with the same thickness, the large-pore (1 mm) structure exhibits a lower Young’s modulus, with 0–78% deformation in the 0–400 kPa pressure range, while the small-pore (0.4 mm) structure demonstrates a higher Young’s modulus, with 0–57% deformation in the same pressure range. Furthermore, when the overall thickness of the sensor is kept constant, increasing the thickness ratio of the small-pore (0.4 mm) composite layer results in a higher Young’s modulus. Conversely, decreasing the thickness ratio of the small-pore (0.4 mm) composite layer leads to a lower Young’s modulus. Therefore, it can be concluded that the dual-layer porous structure plays a significant role in modulating the sensor’s stress–strain performance, which, in turn, affects the sensor’s sensitivity and sensing range. The thickness tunability of the double-layer porous structure provides the sensor with outstanding performance, maintaining high sensitivity across a wide pressure working range, including a maximum sensitivity of $0.063\;{\mathrm{kPa}}^{-1}$ in the 0–23.6 kPa range and a pressure response range of up to 0–654 kPa. To demonstrate the high sensitivity of the sensor within this pressure range, several experiments were designed.

Figure 7b shows the continuous 40 s compression-release tests of the porous composite sensor at various pressures (100 Pa, 500 Pa, 5 kPa, 50 kPa, 150 kPa, and 300 kPa). The relative resistance change in the sensor increases progressively, indicating its ability to differentiate between various pressure levels. Figure 7d presents the continuous 40 s compression-hold tests of the porous composite sensor at different pressures (0 Pa, 500 Pa, 1 kPa, 10 kPa, 20 kPa, and 100 kPa).

The results show that the sensor maintains stable resistance under prolonged pressure, proving its excellent performance stability. In addition to superior sensitivity and a wide sensing range, a good sensor must also have fast response and recovery times. Figure 7c demonstrates the sensor’s fast response time (128 ms) and recovery time (158 ms), enabling its application in smart scenarios such as human–machine interfaces, which require rapid signal acquisition. Under a strain of 40%, the sensor underwent fatigue stability tests exceeding 10,000 cycles, as shown in Figure 7e, demonstrating excellent performance stability. This indicates significant mechanical durability, providing crucial assurance for its practical application.

### 3.4. Application of BLPS Sensors

**Figure 8:**
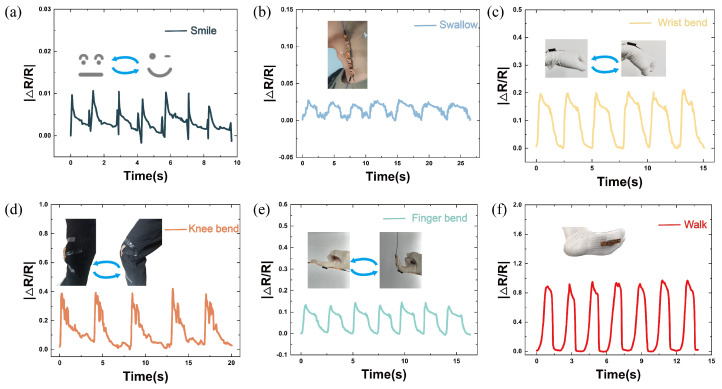
Relative resistance change response of the BLPS sensor under different human movement scenarios: (**a**) Repeated facial expression changes from neutral to smiling. (**b**) Repeated up–and–down movement of the Adam’s apple during swallowing. (**c**) Repeated wrist movement from horizontal to bent. (**d**) Repeated finger extension and bending. (**e**) Repeated knee movement from straight to bent. (**f**) Repeated pressing of the sensor during walking.

The sensor based on a double-layer porous structure offers excellent tunable sensitivity, a wide response range, a fast response time, and outstanding fatigue stability, making it highly suitable for various human motion detection scenarios. To further evaluate its detection capability under different exercise intensities, we attached the sensors with different thickness ratios of the double-layer porous structure to key areas of the human body and monitored the real-time resistance response of the sensor to human movement, as shown in Figure 8.

Figure 8a,b show the resistance changes of the sensor (with a 3:1 thickness ratio of 1 mm and 0.4 mm pore layers) when capturing subtle facial changes during smiling and swallowing, demonstrating the sensor’s application value under low-stress conditions. This also highlights its potential for future applications in facial expression monitoring, as well as in detecting speech and swallowing disorders. Figure 8c–e show the resistance changes in the sensor (with a 1:1 thickness ratio of 1 mm and 0.4 mm pore layers) during finger, wrist, and knee joint bending, indicating that the sensor can still function stably under moderate stress, making it suitable for human joint motion monitoring. Figure 8f illustrates the resistance changes in the sensor (with a 1:3 thickness ratio of 1 mm and 0.4 mm pore layers) under high-stress conditions (such as during gait analysis), showing significant resistance fluctuations, which suggests that the sensor has broad application potential in scenarios involving high stress, such as gait analysis and posture evaluation.

**Figure 9:**
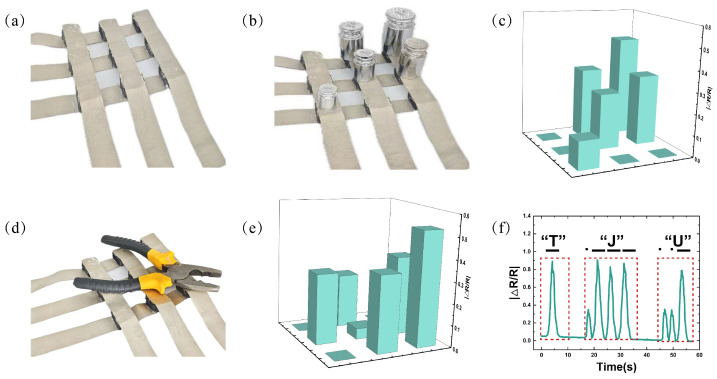
BLPS sensor matrix testing and Morse code pressing test: (**a**) Physical image of the BLPS sensor matrix (3 × 3). (**b**) Placement of different weights on the sensor array (1 × 200 g, 2 × 100 g, 1 × 50 g, and 1 × 20 g weights). (**c**) Relative resistance change response when different weights are placed on the sensor array. (**d**) Placement of pliers on the sensor array. (**e**) Relative resistance change response when pliers are placed on the sensor array. (**f**) Relative resistance change response during simulated Morse code pressing of the letters “T”, “J”, and “U”.

Additionally, a 3 × 3 sensor array was fabricated, as shown in Figure 9a, to simulate the functionality of electronic skin. Figure 9b illustrates the placement of various weights on the sensor array, including one 200 g weight, two 100 g weights, one 50 g weight, and one 20 g weight. Figure 9d shows the placement of a pair of pliers on the sensor array. Figure 9c,e present the resistance responses of the sensor array when different objects were placed on its surface. The results indicate that the sensor array is capable of distinguishing pressure distribution across multiple pixels, demonstrating its potential for use in electronic skin applications.

Morse code is a signal code consisting of intermittent signals that represent different letters, numbers, and punctuation marks through various arrangements. In this study, high stress was used to represent the long signals in Morse code, while low stress was used to represent the short signals. This approach successfully encoded the letters “T”, “J”, and “U” in Morse code, as shown in Figure 9f. These results suggest that the sensor also holds potential for practical application in communication systems.

## 4. Conclusions

In summary, we propose a novel flexible piezoresistive pressure sensor with a dual-pore structure based on a PDMS substrate. The sensor was fabricated using a simple clamp-pressing method and a sacrificial template method with sucrose sugar balls as the sacrificial material. Two layers of CNTs/PDMS sponges with different thicknesses and pore sizes were integrated. By adjusting the thicknesses of the two layers’ pore structures, we successfully achieved adjustable sensitivity and a wide pressure-sensing range for the sensor. The maximum sensitivity within the range of 0–23.6 kPa was $0.063\;{\mathrm{kPa}}^{-1}$, and the pressure response range reached up to 0–654 kPa. Moreover, the sensors fabricated under identical experimental conditions showed highly consistent pore sizes, demonstrating excellent performance consistency, fast response times (128 ms), fast recovery times (158 ms), and outstanding fatigue stability (>10,000 cycles). Under alternating stress and compression, the sensor accurately distinguished between different pressure levels. These excellent piezoresistive properties enable the sensor to monitor real-time human facial expression changes, swallowing actions, joint bending motions, matrix measurements, and Morse code communication in various practical scenarios. The dual-pore structure adjustable pressure sensor proposed in this study provides new insights for electronic product design and shows promising application prospects.

## Data Availability

Data are contained within this article.
